# Performance-Based Specifications of Workability Characteristics of Prestressed, Precast Self-Consolidating Concrete—A North American Prospective

**DOI:** 10.3390/ma7042474

**Published:** 2014-03-27

**Authors:** Wu-Jian Long, Kamal Henri Khayat, Guillaume Lemieux, Soo-Duck Hwang, Ning-Xu Han

**Affiliations:** 1 Guangdong Provincial Key Laboratory of Durability for Marine Civil Engineering, College of Civil Engineering, Shenzhen University, Shenzhen 518060, Guangdong, China; 2 Faculty of Civil, Architectural and Environmental Engineering, Missouri University of Science and Technology, Rolla, MO 65409, USA; E-Mail: khayatk@mst.edu; 3 Faculty of Civil Engineering, Université de Sherbrooke, Sherbrooke, QC J1K 2R1, Canada; E-Mails: glemieux@cement.ca (G.L.); soo-duck.hwang@usherbrooke.ca (S.-D.H.)

**Keywords:** mixture proportioning, workability, rheology, self-consolidating concrete, test method, prestressed concrete

## Abstract

Adequate selection of material constituents and test methods are necessary for workability specifications and performance of hardened concrete. An experimental program was performed to evaluate the suitability of various test methods for workability assessment and to propose performance specifications of prestressed concrete. In total, 33 self-consolidating concrete (SCC) mixtures made with various mixture proportioning parameters, including maximum size and type of aggregate, type and content of binder, and w/cm were evaluated. Correlations among various test results used in evaluating the workability responses are established. It is recommended that SCC should have slump flow values of 635–760 mm. To ensure proper filling capacity greater than 80%, such concrete should have a passing ability that corresponds to L-box blocking ratio (h_2_/h_1_) ≥ 0.5, J-Ring flow of 570–685 mm, slump flow minus J-Ring flow diameter ≤75 mm. Moreover, Stable SCC should develop a column segregation index lower than 5%, and rate of settlement at 30 min of 0.27%/h for SCC proportioned with 12.5 or 9.5 mm MSA. It is recommended that SCC should have a plastic viscosity of 100–225 Pa·s and 100–400 Pa·s for concrete made with crushed aggregate and gravel, respectively, to ensure proper workability.

## 1. Introduction

### 1.1. Workability of SCC

Self-consolidating concrete (SCC) is a relative new class of high-performance concrete that is able to flow and consolidate under its own weight, completely fill the formwork even in the presence of dense reinforcement, whilst maintaining homogeneity, and without the need for any additional compaction [1,2]. SCC mixtures designated for prestressed applications should be highly workable to flow easily through restricted spacing and completely encapsulate reinforcements without any mechanical vibration [2].

Key workability characteristics of SCC can be described in terms of filling ability, passing ability, and stability [3,4]. There are two types of stability characteristics: dynamic and static stability [5,6]. Dynamic stability describes the resistance of the concrete to the separation of the constituents during transport, placement, and spread into the formwork [7]. Static stability refers to the resistance of the concrete to bleeding, segregation, and surface settlement after casting until the beginning of setting [3]. SCC should exhibit high filling ability, proper passing ability, and adequate segregation resistance. These properties are affected by the materials selection, proportioning of materials, admixtures, and application type.

### 1.2. Test Methods and Performance Specifications for SCC

When optimizing the SCC mixture, it is important to select appropriate test methods to qualify the performance of the concrete in the laboratory and later on to control the quality of the concrete at the plant. Various test methods have been used to assess key workability characteristics of SCC. In general, these methods include the components required for evaluating simultaneously deformability, passing ability, and resistance to segregation, since these properties are rather interrelated. The most promising test methods that are relevant for the fabrication of precast, prestressed concrete structural elements are given in Table 1 [2,8–11]. It is also important to note that the slump flow [12], J-Ring [13], and column segregation tests [14] have been standardized by ASTM for use in SCC technology.

The caisson test is used to measure the filling capacity of SCC with a maximum size of aggregate (MSA) of 20 mm. This test can be used in the design of the mixture whenever such conditions are presented for precast, prestressed elements. It is recommended to complement the Visual Stability Index (VSI) test with a quantitative test, such as the column segregation or surface settlement test. The former is rather long as it involves the determination of the relative coarse aggregate content at four sections along a concrete column. For field use, this may be substituted by determining the relative coarse aggregate content at the top and bottom sections only. The surface settlement test is rather long for use as a quality control test in the field. Instead, the rate of settlement after 30 min can be determined.

The various deformability and stability test results can be related to the rheological parameters of the concrete, yield stress and plastic viscosity [15,16]. For example, the slump flow and T-50 values can be related to the yield stress and plastic viscosity, respectively. Similarly, the speed of spread of the concrete through the V-funnel and L-box can be related to the plastic viscosity [17]. Assaad *et al.* [18] related the segregation index (Iseg) of the column segregation test to the apparent yield stress (g) and torque plastic viscosity (h) determined using the IBB rheometer. SCC with apparent g and h of 0.3–1.7 N.m and 17–30 N.m.s, respectively, were shown to develop adequate resistance to segregation with Iseg values of 2%–4%.

Khayat *et al.* [17] found that SCC with g values ranging from 0.3 to 1.7 N.m and h values of 17–27 N.m.s can achieve high passing ability determined using the L-box with flow time of 4–8 s. Better understanding of the rheological parameters that control the workability of SCC is therefore important in developing mix design approaches and interpreting quality control test methods.

## 2. Experimental Program

### 2.1. Materials

Two types of Portland cement (Type MS and Type HE, Cement Quebec, Quebec, Canada) and two supplementary cementitious materials (blast-furnace slag and Class F fly ash) were used. The physical properties and chemical composition of cement and supplementary cementitious materials are presented in Table 2. Three types of crushed aggregates corresponding to MSA of 19 mm, 9.5 mm, 12.5 mm and one type of gravel with MSA of 12.5 mm were selected. The aggregates conform to AASHTO T 27 specifications. Natural siliceous sand with a specific gravity of 2.66 conforming to AASHTO T 27 specifications was used. The grading and properties of the various aggregate and sand are summarized in Table 3. The particle-size distributions of the aggregate combinations are within the AASHTO recommended limits. Polycarboxylate-based high-range water-reducing admixture (HRWRA) complying with AASHTO M 194, Type F was used. An air-entraining admixture (ASTM C 260) was incorporated to obtain an initial air content of 4%–7% in selected SCC mixtures. An organic, thickening-type VMA representative of products commonly used in the precast industry was used for the experimental program.

It is important to note that the research work presented herein is part of the NCHRP-Project conducted by the Transportation Research Board in the USA. The objective of this research is to investigate the effect of mixture proportioning and material characteristics on key parameters which can affect fresh properties of SCC, and to develop the specifications for the use of SCC in precast, prestressed applications, including recommended changes to the AASHTO LRFD Specifications.

After consultation with various precasting plants across the U.S. who are using SCC and admixture suppliers, it was indicated that Type I (MS), Type III (HE), and Type II cements are employed, in order of importance, for producing SCC for the casting of prestressed beams, as well as precast architectural panels and precast products. The survey also indicated that fly ash and blast-furnace slag are used with substitution rates of up to 40% and 50%, respectively, of the total binder content. Moreover, the survey indicated that crushed limestone aggregate is widely used in precasting operations with nominal aggregate sizes mostly of 19 mm and 9.5 mm, and in some cases 12.5 mm. Of particular interest to precast, prestressed applications is the use of polycarboxylate-based HRWRA to reduce water demand, typically without secondary effect on set retardation. The use of this class of HRWRA could also potentially reduce or eliminate heat curing.

In this investigation, three different binder compositions corresponding to Type I/II (MS) cement made without any supplementary cementitious materials and Type III (HE) cement employed with either 30% blast-furnace slag replacement or 20% Class F fly ash replacement were considered. Such blast-furnace slag and fly ash substitutions are typical of values used in some regions in the United States where such supplementary cementitious materials are employed to reduce heat rise and the cost of concrete while enhancing its durability.

### 2.2. Mixture Composition and Mixing Sequence

Prior to undertaking the experimental program, a detailed literature review was conducted to review the state of the art pertaining to the proportioning and performance of SCC designated to prestressed, precast applications. The results of this literature review and the survey conducted with precast producers in the U.S. were taken into consideration in finalizing the research program and selecting the test parameters. In this investigation, the ranges of the mix design parameters are selected to cover a wide scope of mixture formulations and material characteristics used in precasting plants in the U.S. Based on vast preliminary laboratory tests, a parametric study which including 33 typical precast, prestressed SCC mixtures was then undertaken to evaluate the influence of the type and nominal size of coarse aggregate, binder type, and w/cm on key workability characteristics, stability, and rheological parameters of SCC.

As presented in Table 4, 24 non-air entrained SCC mixtures (No. 1–24) were prepared to evaluate workability of SCC. The mixtures were prepared using either crushed aggregate or gravel with MSA of 19, 12.5, and 9.5 mm, w/cm of 0.33 and 0.38, and three binder compositions: Type MS cement as well as Type HE cement containing either 30% slag or 20% Class F fly ash replacement of the total binder content. Three air-entrained SCC (No. 25–27) with low w/cm were also investigated. SCC mixtures were proportioned with 460 and 480 kg/m^3^ of binder. The HRWRA dosage was adjusted to achieve an initial slump flow of 680 ± 20 mm.

Three SCC mixtures (No. 28–30) with relatively low slump flow of 620 ± 20 mm similar to mixtures No. 1–3 and three other mixtures (No. 31–33) with high slump flow values of 735 ± 25 mm similar to mixtures No. 4–6 were prepared to evaluate the effect of mixture deformability on workability.

The SCC mixtures were prepared in 110-L batches using a drum mixer. The mixing sequence consisted of wetting the sand and coarse aggregate with half of the mixing water, followed by the addition of the binder. The HRWRA and VMA diluted with the remaining mixing water were then introduced over 30 s, and the concrete was mixed for 2.5 min. The concrete remained at rest in the mixer for 2 min for fluidity adjustment and to enable any large air bubbles entrapped during mixing to rise to the surface. The concrete was then remixed for 3 min. The fresh properties of SCC were measured at 10 and 40 min after cement and water contact.

## 3. Results and Discussion

### 3.1. Combined Test Methods to Evaluate Filling Capacity

The workability responses obtained in the parametric investigation are used to compare the various test responses for SCC and highlight advantages and limitations of these test methods. In general, test methods used to evaluate workability of SCC provide one workability index, which is not sufficient to adequately describe the flow behaviour of SCC. Therefore, proper combinations of various test methods can be employed to facilitate the assessment of workability and improve the quality control procedure of SCC.

The workability test results in the SCC investigation were used to derive multi-regression equations relating the filling capacity of SCC with slump flow, J-Ring flow, and L-box blocking ratio (h_2_/h_1_), as shown in Table 5. The multiple regressions were established using workability data determined shortly after the end of mixing (10 min of age).

**Figure 1.**
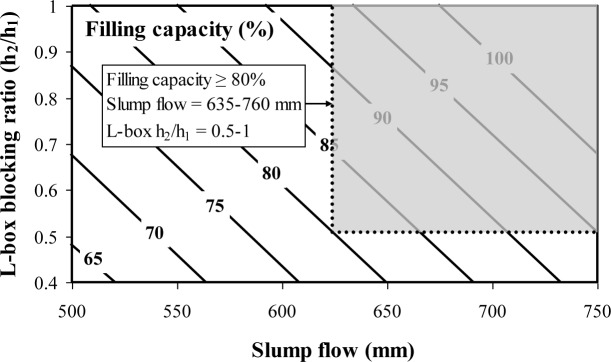
Contour diagrams between filling capacity, slump flow, and L-box blocking ratio determined at 10 min (R^2^ = 0.82) (Equation (1)).

Contour diagrams of the filling capacity values as function of the slump flow and L-box blocking ratio are plotted in Figure 1 based on the multiple regression correlation given in Equation (1). For a given lower limit of blocking ratio of 0.5, the increase in slump flow from 635 to 760 mm can be expected to increase the filling capacity from 80% to 95%.

**Figure 2.**
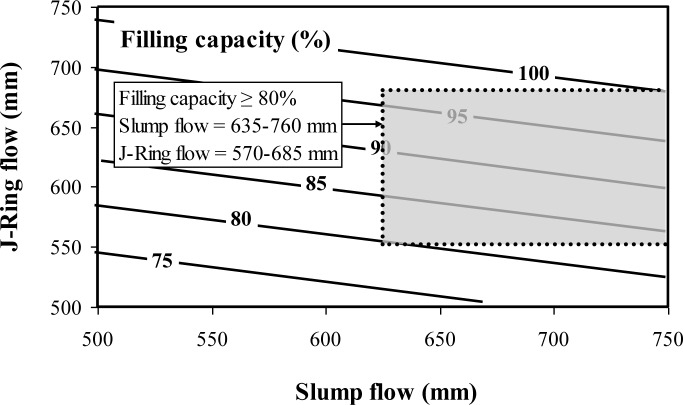
Contour diagrams between filling capacity, slump flow, and J-Ring flow determined at 10 min (R^²^ = 0.83) (Equation (2)).

**Figure 3.**
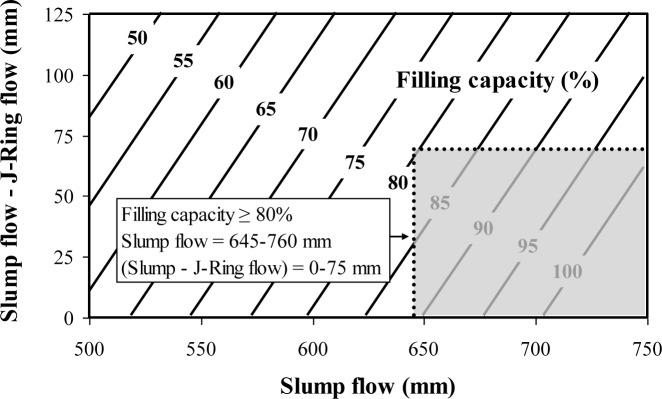
Contour diagrams between filling capacity, slump flow, and (slump flow-J-Ring flow) determined at 10 min (R^²^ = 0.80) (Equation (3)).

The multiple regression correlations given in Equations (2) and (3) are used also to establish contour diagrams of the filling capacity of SCC as function of slump flow and J-Ring flow (Figure 2) and slump flow and the spread between slump flow and J-Ring flow values (Figure 3). As can be observed from Figure 6 and for a given slump flow, a decrease in J-Ring spread can lead to a decrease in filling capacity. On the other hand and for a given slump flow, an increase in the difference between slump flow and J-Ring values can lead to a reduction in filling capacity resulting from some lack in the restricted deformability across closely spaced obstacles (Figure 3).

### 3.2. Evaluation of Static Stability

The surface settlement test involves the determination of the capacity of the concrete to undergo a settlement due to segregation and consolidation of the plastic concrete. Consolidation can occur due to movement of entrapped or entrained air or bleed water to the top surface. Surface settlement can take place in the absent of segregation, which implies separation between coarse aggregate and mortar. Based on *in-situ* compressive strength and pull-out bond strength results obtained from NCHRP project 18–12, SCC mixtures having maximum surface settlement of 0.5% are found to have more homogenous *in-situ* properties than those of vibrated HPC mixtures [8].

**Figure 4.**
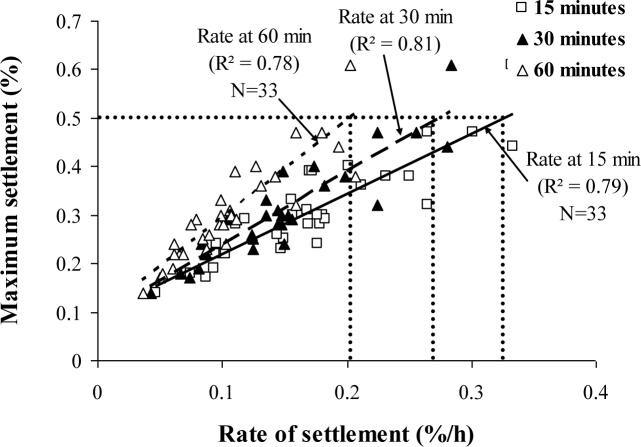
Relationship between rate of settlement and maximum settlement.

In addition to determining the maximum surface settlement, the initial rate of settlement (expressed as relative settlement per hour) can be calculated as follows:

Settlement rate (%per hour)={St(%)−St−5(%)}/{5(min)/60(min)} (5)

where, St is settlement value at a given time (min) *t*, St-5 is the settlement value at time of *t* minus 5 min. Settlement rates determined at 15, 30, and 60 min after the beginning of surface settlement testing are correlated to the maximum settlement values in Figure 4. The 30-min rate of settlement, which is calculated as the spread in settlement values between 25 and 30 min divided by 5 min, can be used to speed up surface settlement testing. A settlement rate of 0.27% per hour after 30 min is shown to correspond to the upper limit of 0.5% maximum settlement. The test period can even be reduced to 15 min, as in the case of the column segregation test (ASTM C 1610). In this case, a settlement rate of 0.32% per hour after 15 min can correspond to the upper limit of 0.5% maximum settlement.

**Figure 5.**
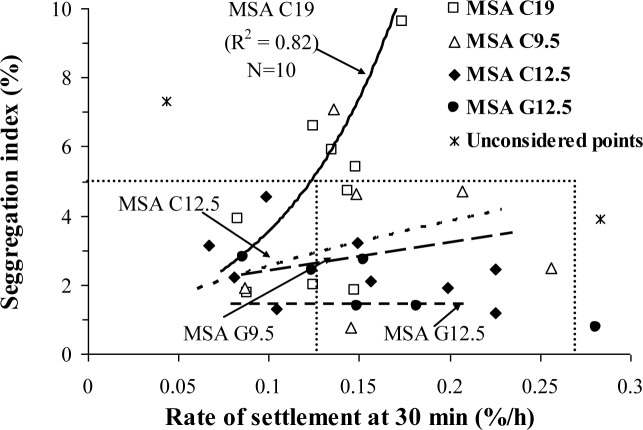
Variation in column segregation index with rate of surface settlement.

The rates of settlement at 30 min are compared in Figure 5 to the segregation index determined from the column segregation test. The latter test involves casting the concrete in an experimental column to determine the distribution of coarse aggregate along the column after 15 min of rest. For the column segregation test, the relative coarse aggregate portions in four sections along the experimental column are determined to calculate the C.O.V. (or the segregation index). This is in contrast with the evaluation of coarse aggregate contents from the top and bottom sections of the column, as per ASTM C 1610. These two values are used to determine the resistance to segregation, as follows:

$$Percent\;static\;segregation(\%)=2\times \left(\frac{CA_{B}-CA_{T}}{CA_{B}+CA_{T}}\right)\times 100$$ (6)

where CAT and CAB refer to the mass of coarse aggregate at the top and bottom sections, respectively.

Regardless of the rate of settlement, no significant spread was found in the column segregation index (C.O.V.) for the SCC mixtures proportioned with 12.5 or 9.5 mm MSA. It is important to note that the majority of SCC mixtures tested were highly stable with surface settlements lower than 0.5%, rates of settlement at 30 min lower than 0.27%/h, and column segregation indices lower than 5%.

The segregation index of SCC made with 19 mm MSA is shown to increase with the increase in the rate of settlement. Given the correlations shown in Figure 5, stable SCC proportioned with 19 mm MSA should have rates of settlement lower than 0.12%/h in order to secure a segregation index lower than 5%.

The MSA and type of coarse aggregate have therefore considerable influence on the column segregation index. For a given rate of settlement, SCC proportioned with gravel with 12.5 mm MSA exhibited lower segregation index (*i.e.*, more stable) compared to similar concrete made with crushed aggregate of the same MSA. Similarly, for a given settlement rate, SCC made with 12.5 or 9.5 mm MSA had lower segregation index compared to those prepared with 19 mm MSA.

**Figure 6.**
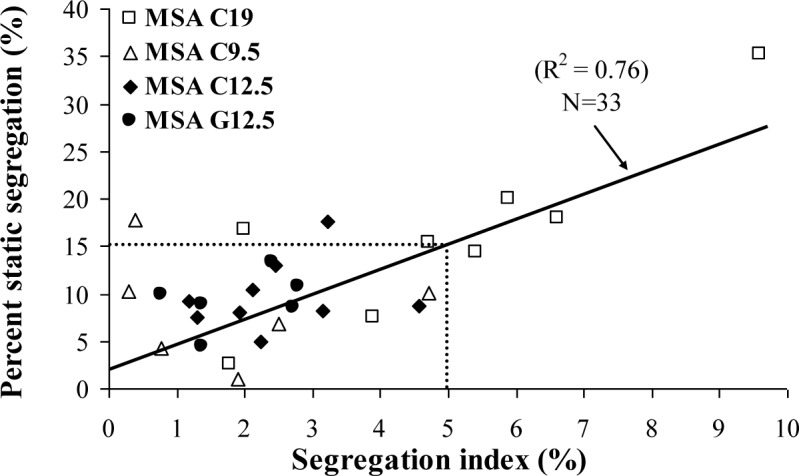
Relationship between column segregation index (C.O.V.) and percent of static of segregation (S).

As illustrated in Figure 6, good correlation coefficient (R^2^ = 0.76) can be established between the segregation index (determined from four sections along the segregation column) and the percent static segregation (S) (determined only from the top and bottom sections of the segregation column), regardless of the MSA in use. On the average, stable SCC should have a static segregation lower than 15% to maintain a maximum segregation index of 5%.

### 3.3. Correlations between Rheological Parameters and Workability Test Results

The rheological measurement of the 33 SCC mixtures investigated in parametric study was performed using a modified Tattersall two-point workability rheometer (MK III model) [19–21]. The Bingham model can be expressed as a function of the shear rate (γ);τ = τ_0_ + μ_p_γ with τ_0_ and μ_p_ corresponding to the yield stress and plastic viscosity, respectively. The yield stress values ranged between 10 and 130 Pa for SCC with slump flow values of 640–690 mm. The plastic viscosity values ranged between 75 and 700 Pa·s, with the majority of plastic viscosity results being within the 100 and 500 Pa·s range.

The rheological parameters for the SCC mixtures were compared to the various workability test results to identify combinations of rheological parameters necessary to provide adequate filling ability, filling capacity, and stability of SCC for successful casting of prestressed elements. Relatively high degree of scattering was observed for either relationship, which could be due to differences in mixture proportioning and MSA that affect the flow velocity of the SCC.

**Figure 7.**
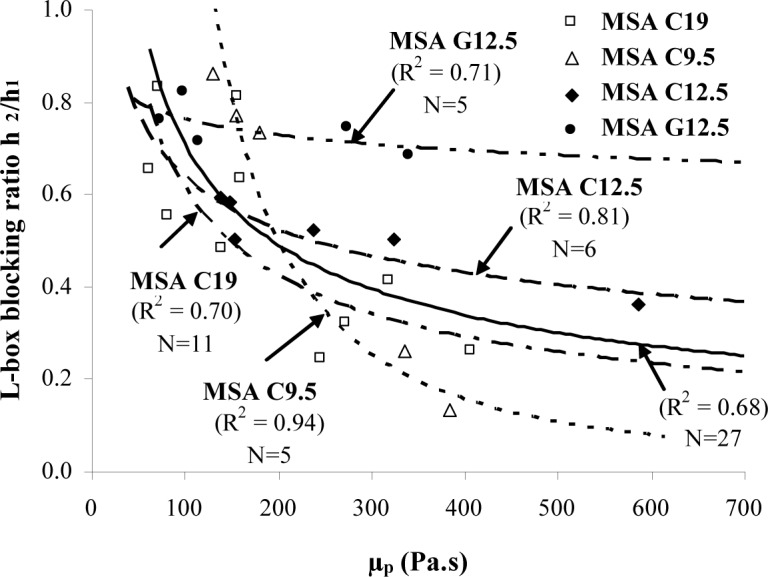
Relationship between plastic viscosity and L-box blocking ratio.

Among the various workability responses, the L-box blocking ratio (h_2_/h_1_) exhibited good correlation with the plastic viscosity providing that the comparison is performed for each aggregate separately. As presented in Figure 7, the h_2_/h_1_ ratio decreases with the increase in plastic viscosity. Viscous concrete can then hinder the flow and lead to low L-box blocking ratio. Depending on the aggregate characteristics, the plastic viscosity should be limited to 200 Pa·s in order to secure a minimum blocking ratio of 0.5–0.7. The lower limit 200 Pa·s can be increased to 225 Pa·s for SCC made with coarse aggregate of 12.5 mm MSA.

**Figure 8.**
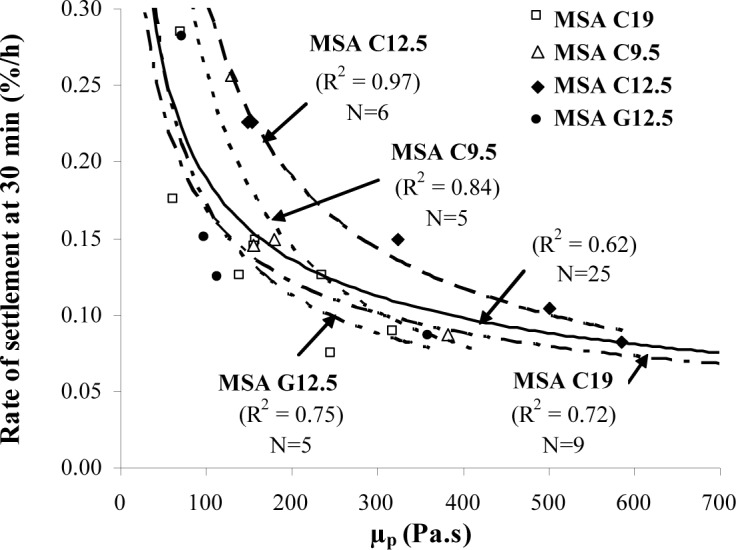
Variations of the rate of surface settlement determined after 30 min of testing and initial plastic viscosity for SCC made with different coarse aggregates.

As shown in Figure 8, the rate of settlement determined after 30 min can be correlated to the initial plastic viscosity. According to Hwang *et al.* [22], SCC can be considered to have high level of static stability when the maximum surface settlement is 0.5% and the rate of settlement after 30 min is limited to 0.16%/h. This latter limit can be achieved when the SCC has a minimum initial plastic viscosity of 150 Pa·s.

**Figure 9.**
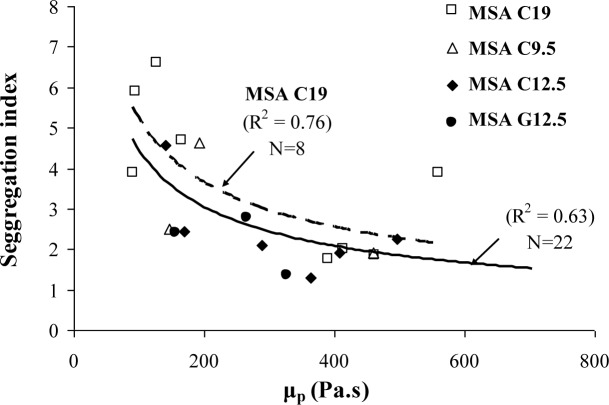
Variations of the segregation index and plastic viscosity determined at 10 min.

The segregation index results determined from the column segregation test are compared to the plastic viscosity results determined at 10 min in Figure 9. An increase in plastic viscosity can lead to lower segregation coefficient. For a given level of viscosity, SCC made with the larger MSA can undergo greater risk of segregation. For a given upper limit of segregation coefficient (for example 4%), SCC made with crushed aggregate with MSA of 9.5 mm or crushed aggregate or rounded gravel with MSA of 12.5 mm should have a minimum plastic viscosity of 150 Pa·s. This limit should be increased to 200 Pa.s for SCC made with crushed coarse aggregate of 19 mm MSA.

### 3.4. Recommended Test Methods and Specifications

The use of proven combinations of test methods in precast, prestressed applications is necessary to reduce time and effort required for quality control of SCC at the precasting plant. A caisson filling capacity value of 80% is considered as a lower limit for casting of densely reinforced sections, typically found in precast, prestressed applications. The L-box blocking ratio (h_2_/h_1_) index, J-Ring flow, or the spread between the slump flow and J-Ring flow can be combined with the slump flow to evaluate the filling capacity of SCC. The recommend combined test methods for evaluating the filling capacity of SCC are:

- Combined test methods-I: Slump flow and L-box blocking ratio (h_2_/h_1_);
- Combined test methods-II: Slump flow and J-Ring flow.

Table 6 presents a set of performance specifications of SCC that can be used in prestressed applications. Such specifications correspond to SCC with slump flow of 635–760 mm and, depending on the passing ability test in use, L-box blocking ratio (h_2_/h_1_) greater than 0.5, J-Ring flow of 570–685 mm, and a spread in slump flow and J-Ring flow values lower than 75 mm. As indicated in Table 6, regardless of the MSA, stable SCC should develop a column segregation index (C.O.V.) and percent static segregation (S) lower than 5% and 15%, respectively. The recommended limits for surface settlement depend on the MSA. SCC proportioned with 19 mm and 12.5 or 9.5 mm MSA should have maximum rates of settlement at 30 min of 0.12%/h and 0.27%/h, respectively.

In the case of plastic viscosity, it is recommended that SCC made with crushed aggregate have a plastic viscosity of 100–225 Pa·s to ensure adequate passing ability and static stability. This range can be from 100 to 400 Pa·s for SCC made with gravel having 12.5 mm MSA. The lower limit of 100 Pa·s is required for static stability; maximum rate of settlement at 30 min of 0.27%/h and a maximum column segregation index (C.O.V.) of 5%, regardless of the aggregate type. The upper limit of plastic viscosity of 225 and 400 Pa·s is necessary for SCC with slump flow consistency of 660–700 mm to achieve adequate passing ability (minimum L-box blocking ratio of 0.5).

## 4. Conclusions

Based on the test results and discussion above, the following conclusions can be drawn:

- Performance-based specifications are suggested for high-performance SCC designated for the filling of restricted sections typically found in prestressed applications. Instead of testing the filling capacity of concrete by using the caisson test, a combination of passing ability and non-restricted deformability can be used to assess the filling capacity of SCC;
- A combination of the slump flow and either the L-box blocking ratio (h_2_/h_1_), J-Ring flow can be used to assess filling capacity of SCC for quality control and design of SCC for placement in restricted sections, typically in prestressed applications;
- SCC designated for prestressed applications should have a slump flow of 635–760 mm, an h_2_/h_1_ value higher than 0.50 (laboratory use), a J-Ring flow of 570–685 mm (plant use), a spread between slump flow and J-Ring flow lower than 75 mm (plant use), and a caisson filling capacity value greater than 80%;
- Regardless of the MSA, stable SCC should develop a column segregation index (C.O.V.) and percent static segregation (S) lower than 5% and 15%, respectively;
- The recommended limits for surface settlement depend on the MSA. SCC proportioned with 19 mm and 12.5 or 9.5 mm MSA should have maximum rates of settlement at 30 min of 0.12%/h and 0.27%/h, respectively;
- It is recommended that SCC made with crushed aggregate have a plastic viscosity of 100–225 Pa·s to ensure adequate passing ability and static stability. This range can be from 100–400 Pa·s for SCC made with gravel having 12.5 mm MSA.

## Figures and Tables

**Table 1.** Test methods relevant for fabrication of precast, prestressed structural elements.

Workability	Test methods for mix design	Test methods for quality control at plant
Filling ability	Slump flow T-50_cm_ slump flow	Slump flow T-50_cm_ slump flow
Passing ability	L-box J-Ring V-funnel	L-box J-Ring
Filling capacity	Caisson test (filling vessel)	–
Segregation resistance	VSI Surface settlement and rate of settlement Column segregation	VSI Rate of settlement after 30 min Column segregation
Rheology	Modified Tattersall two-point workability rheometer	–

**Table 2.** Physical properties and chemical composition of cement and supplementary cementitious materials.

Cement and supplementary cementitious materials	Type MS cement	Type HE cement	Class F fly ash	Blast-furnace slag
*Physical properties*				
Specific gravity	3.14	3.15	2.53	2.95
Blaine specific surface area, m^2^/kg	390	530	410	400
Passing No. 325 (45 μm), %	91	99	90	92

*Chemical composition*, %				

SiO_2_	21.4	20.0	52.4	36.0
Al_2_O_3_	4.6	5.4	27.2	10.4
Fe_2_O_3_	2.9	2.3	8.3	1.5
CaO	63.3	63.5	4.5	42.9
MgO	2.0	1.4	0.96	6.7
SO_3_	3.4	4.4	0.05	0.48
K_2_O	0.94	1.1	2.33	0.37
Na_2_O	0.07	0.15	0.20	0.17
Na_2_O eq*	0.69	0.88	1.74	0.41
LOI	0.98	0.80	2.73	0.41

* Na_2_O equivalent = Na_2_O + 0.64 K_2_O.

**Table 3.** Grading and properties of coarse aggregate and sand.

Sieve opening	Siliceous sand	Crushed coarse aggregate			Gravel
	0–4.75 mm	19–4.75 mm	12.5–4.75 mm	9.5–2.36 mm	12.5–4.75 mm
25 mm	100	100	100	100	100
19 mm	100	99	100	100	100
12.5 mm	100	68	95	100	99
9.5 mm	100	40	69	100	94
4.75 mm	98	7	18	13	32
2.36 mm	85	1	4	2	1
1.18 mm	72	1	3	2	–
600 μm	55	–	–	–	–
300 μm	32	–	–	–	–
150 μm	9	–	–	–	–
Pan	2	0	0	0	0
Specific gravity	2.66	2.72	2.71	2.73	2.66
Absorption, %	1.12	0.31	0.44	0.38	1.26

**Table 4.** Parametric experimental program.

Type	Mixture No.	Aggregate Type and MSA				Type and Content of Binder			w/cm	

		Crushed 19 mm	Crushed 9.5 mm	Crushed 12.5 mm	Gravel 12.5 mm	Type MS 480 kg/m^33^	Type HE + 30% Slag 460 kg/m^3^	Type HE + 20% fly ash 460 kg/m^3^	0.33	0.38
**Non air-Entrained (AEA) concrete**	1	x				x			x	
	2	x					x		x	
	3	x						x	x	
	4	x				x				x
	5	x					x			x
	6	x						x		x
	7		x			x			x	
	8		x				x		x	
	9		x					x	x	
	10		x			x				x
	11		x				x			x
	12		x					x		x
	13			x		x			x	
	14			x			x		x	
	15			x				x	x	
	16			x		x				x
	17			x			x			x
	18			x				x		x
	19				x	x			x	
	20				x		x		x	
	21				x			x	x	
	22				x	x				x
	23				x		x			x
	24				x			x		x
**AEA**	25–27	• Air entrainment of 4%–7% and slump flow of 680 ± 20 mm								
		• 0.33 w/cm, Type HE + 20% Class F fly ash, crushed aggregate MSA of 12.5 mm								
**Non AEA Concrete**	28–30	• Low filling ability, slump flow of 620 ± 20 mm								
		• 0.33 w/cm, Type HE + 30% slag, crushed aggregate MSA of 19 mm								
	31–33	• High filling ability, slump flow of 735 ± 25 mm								
		• 0.38 w/cm, Type HE + 30% slag, crushed aggregate MSA of 19 mm								

Sand-to-total aggregate ratio (S/A) is fixed at 0.5, by volume.

**Table 5.** Multi-regression equations.

Workability	Equations	R^2^	No.
Filling capacity (%)	= −9.64 + 0.12 slump flow (mm) + 28.25 h_2_/h_1_	0.82	(1)
	= −32.82 + 0.05 slump flow (mm) + 0.14 J-Ring flow (mm)	0.83	(2)
	= −32.82 + 0.19 slump flow (mm.) −0.14 {Slump flow (mm) − J-Ring flow (mm)}	0.80	(3)
	= 17.45 + 0.09 J-Ring flow (mm) + 19.99 h_2_/h_1_	0.85	(4)

**Table 6.** Combined test methods and recommended workability values for SCC designated for prestressed applications.

Workability	Combined test methods-I	Combined test methods-II
Filling ability	Slump flow: 635–760 mm	
	L-box blocking	J-Ring flow: 570–685 mm
Passing ability	ratio (h_2_/h_1_) ≥ 0.5	Slump flow-J-Ring flow ≤ 75 mm
	Plant and laboratory use	Plant use
Filling capacity	Caisson filling capacity ≥ 80% (laboratory use)	
	Combined test methods (I or II)	
	I. Segregation resistance	
	- Column segregation index (C.O.V.) ≤ 5%	
Static stability	- Percent static segregation (S) ≤ 15%;	
	II. Rate of surface settlement at 30 min	
	- MSA of 9.5 and 12.5 mm ≤ 0.27 (%/h) (Max. settlement of 0.5%)	
	- MSA of 19 mm ≤ 0.12 (%/h) (Max. settlement of 0.3%)	
Rheological parametersto ensure proper workability	Plastic viscosity (μ_p_)	
	- 100–225 Pa·s for SCC made with crushed aggregate	
	- 100–400 Pa·s for SCC made with gravel	
